# Histopathological pattern of Orbito-Ocular Lesions, a retrospective hospital-based study spanning 15 years

**DOI:** 10.4314/ahs.v22i3.23

**Published:** 2022-09

**Authors:** Dumebi H Kayoma, Dele E Imasogie

**Affiliations:** 1 Department of Ophthamology University of Benin, Benin City, Edo State, Nigeria; 2 Department of Anatomic Pathology, University of Benin Teaching Hospital, Benin City, Nigeria

**Keywords:** Ophthalmic biopsies, ophthalmic lesions, malignant orbito-ocular tumours, benign orbito-ocular tumours, degenerative ophthalmic lesion

## Abstract

**Background:**

Many previous studies on orbito-ocular lesions are skewed in favour of the neoplastic lesions in general and the malignant lesions in particular. This, therefore, creates a vacuum on the spectrum of these lesions, thus may result in problematic diagnostic bias by the ophthalmologist and pathologist.

**Objective:**

To give the spectrum and relative frequencies of orbito-ocular biopsies and by extension orbito-ocular lesions/diseases at the University of Benin Teaching Hospital (UBTH).

**Materials and Methods:**

A retrospective descriptive study of all cases of orbito-ocular biopsies with histopathologic diagnosis.

**Results:**

There were 236 orbito-ocular biopsies. The male to female ratio was slightly in favour of the females. Orbito-ocular biopsies had a wide age range that spanned from the 1st to 10th decade, mean age in the 3^rd^ decade (20–29years) and a peak age in the 1st decade (0–9 years). The neoplastic lesions were the prevalent indication for orbito-ocular biopsies (63.72%) while the conjunctiva (58.10%) was the most common site for orbito-ocular biopsies.

**Conclusion:**

This study noted a wide array of orbito-ocular lesions for which biopsies were done for histopathological diagnosis. This we hope will in no small measure increase the diagnostic precision of the ophthalmologist and the pathologists in our own environment.

## Introduction

The orbit is that part of the skull that is formed by 7 bones.^1^ These bones form the orbital cavities that house the eyeballs and its adnexal structures. 1 These structures are home to diseases that vary from the non-neoplastic lesions (degenerative, inflammatory, infective, traumatic) to the neoplastic lesion (benign, premalignant and malignant).^2^ These diseases are a significant cause of morbidity and mortality,^3^, ^4^ however studies on lesions of the eye and its adnexal are mostly on neoplastic lesions, although limited histopathologic studies which include the complete spectrum of ophthalmic biopsies have been carried out.^3^, ^5^–^10^

The rarity at which orbito-ocular lesions occur complicates the recognition of their fine and sometimes subtle presentations.^10^, ^11^ This can pose a huge diagnostic dilemma to the ophthalmologist and pathologist, more so that clinical signs and symptoms of ocular malignancies had been reported to mimic more commonly occurring benign conditions.^10^

To this end, we aim to determine the spectrum of histopathological diagnosis of orbito-ocular lesions at the University of Benin Teaching Hospital (UBTH).

## Materials and Methods

This study was a retrospective chart review of all orbito-ocular biopsies that were received in the Department of Anatomic Pathology, UBTH. The Department of Anatomic Pathology UBTH, Edo State, South-South, Nigeria was the location for this study. This hospital is a referral centre to all other secondary and primary health care facilities within Edo and Delta sub-region and from elsewhere especially its catchment area of Ondo, Kogi and Anambra states.^12^ This study was carried out over a15 year period from from 1st of January 2005 to 31^st^ of December 2019.

Data for this study were obtained from the surgical pathology registers, histology request cards, and duplicate copies of histopathology reports. Information obtained included age, gender, nature of specimen, site of lesion and histopathologic characteristics. The data obtained were analysed using the Statistical Package for Social Sciences, version 20 (SPSS 20, IBM Corp. Armonk, NY, United States of America).

Qualitative (sex, site and histopathological diagnosis) and quantitative (age) data were analysed for discussion. For the former, the respective frequency of orbito-ocular lesions and their corresponding rates in percentages were analysed. For the latter, the age range, mean age, standard deviation, peak age were analysed for the respective orbito-ocular lesions

Ethical approval was sought from the ethics and research committee of the University of Benin Teaching Hospital. It was granted with an assigned ethical approval number, ADM/E22/A/VOL.VII/14831124.

## Results

Two hundred thirty six (236) ophthalmic biopsies were received during the period under review. The age group and sex distribution of the ophthalmic biopsies is as shown in the Table 1. The ages of 27 of the cases were not documented and analyses pertaining to age were done on the documented ages of the remaining 209 cases. Their ages ranged from 6 months to 96 years with a mean age and standard deviation of 26.76 ± 23.74 years, and a median age of 26.00 years. Orbito-ocular biopsies peaked in the 1st decade and accounted for 64 cases (27.12%). This decade also accounted for the peak age in the males and females. A slight female predilection was observed in the study population with a male to female ratio of 1:1.01 with a corresponding percentage of 49.6% (117 male) to 50.4% (118 female). The sex was not specified in a case.

**Table 1:** showing age and sex distribution of orbito-ocular biopsies (lesions)

	sex*		
		
Age group*	Male	Female	Total
0–9	31	33	64
10–19	4	7	11
20–29	14	18	32
30–39	11	15	26
40–49	15	16	31
50–59	14	10	24
60–69	10	3	13
70–79	1	3	4
80–89	3	0	3
90–99	0	2	2

Total	103	107	210

sex* = cases with specified sex and corresponding specified age

Age group* = cases with specified age

The malignant orbital tumours (MOTs) were the most common orbito-ocular lesions as they accounted for 36.0 % of cases while the benign orbito-ocular tumours (BOTs) (26.7%), degenerative lesions (11.0%), and infective/inflammatory lesions (9.0%) were next in frequencies in decreasing order as shown in Tables, 2, 3 and 4. Other less common orbito-ocular lesions are as shown in Table 4.

**Table 2:** Malignant orbito-ocular tumours

Malignant ophthalmic tumours	Frequency	% within MOT	% in Ophthalmic biopsies
Squamous cell carcinoma	39	45.9	16.53
Retinoblastoma	38	44.7	16.10
Melanoma	4	4.7	1.69
Rhabdomyosarcoma	2	2.4	0.84
Kaposi sarcoma	1	1.2	0.42
Adenoid cystic carcinoma	1	1.2	0.42

Total	85	100	36.00

**Table 3:** Benign orbito-ocular tumours

Benign ophthalmic tumours	Frequency	% with BOT	% within Ophthalmic biopsies
Squamous cell papilloma	21	33.3	8.9
Pyogenic granuloma	15	23.8	6.3
Lipoma	8	12.7	3.4
Benign naevocellular lesion	4	6.3	1.7
Apocrine hidrocystoma	3	4.7	1.3
Neurofibroma	2	3.2	0.9
Angiofibroma	2	3.2	0.9
Capillary hemangioma	1	1.6	0.4
Dermatofibroma	1	1.6	0.4
Syringocystadenoma	1	1.6	0.4
Sebaceous adenoma	1	1.6	0.4
Pilocyctic astrocytoma	1	1.6	0.4
Plomophic adenoma	1	1.6	0.4
Verrucae plana	1	1.6	0.4
Conjunctival lymphoma	1	1.6	0.4

**Total**	**63**	**100**	**26.7**

**Table 4:** Showing less common orbito-ocular lesions

Ophthalmic lesions	Frequency	% within individual lesion	% within ophthalmic biopsies
**Premalignant ophthalmic** **lesion**			
Dysplasia of the conjunctiva	14.0	100.0	5.9

**Total**	**14.0**	**100.0**	**5.9**

**Ophthalmic cyst**			
Epidermal inclusion cyst	18.0	94.7	7.6
Lacrimal gland cyst	1.0	5.3	0.4

**Total**	**19.0**	**100.0**	**8.0**

**Infective and Inflammatory** **lesion**			
Pan-ophthalmitis	11.0	52.3	4.7
Chalazion	3.0	14.3	1.3
Staphyloma	3.0	14.3	1.3
Conjunctivitis	2.0	9.5	0.9
Inflammatory hyperplastic polyp	1.0	4.8	0.4
Conjunctiva abscess	1.0	4.8	0.4

**Total**	**21.0**	**100.0**	**9.0**

**Degenerative lesion**			
Pterygium	26.0	100.0	11.0

**Total**	**26.0**	**100.0**	**11.0**

**Others**			
Traumatic eye	5.0	62.5	2.1
Necrosis	2.0	25.0	0.9
Choriostoma	1.0	12.5	0.4

**Total**	**8.0**	**100.0**	**3.4**

**Grand Total**	**88.0**	**-**	**37.3**

**Figure 1:**
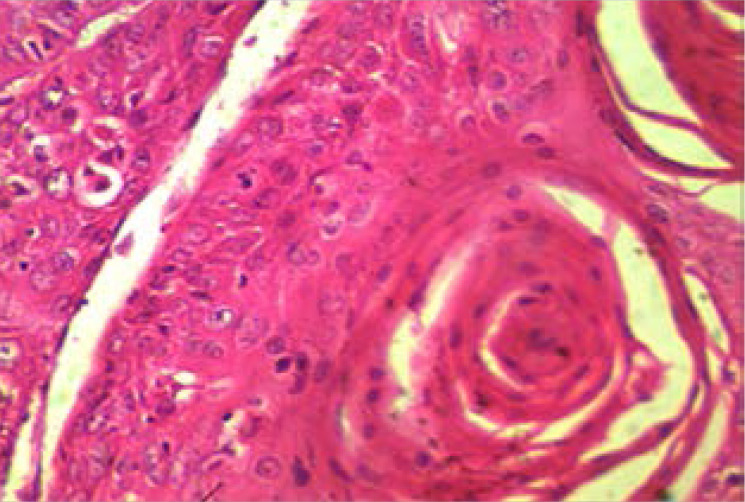
Photomicrograph showing well differentiated squamous cell carcinoma (Haematoxylin and Eosin stains, X400).

**Figure 2:**
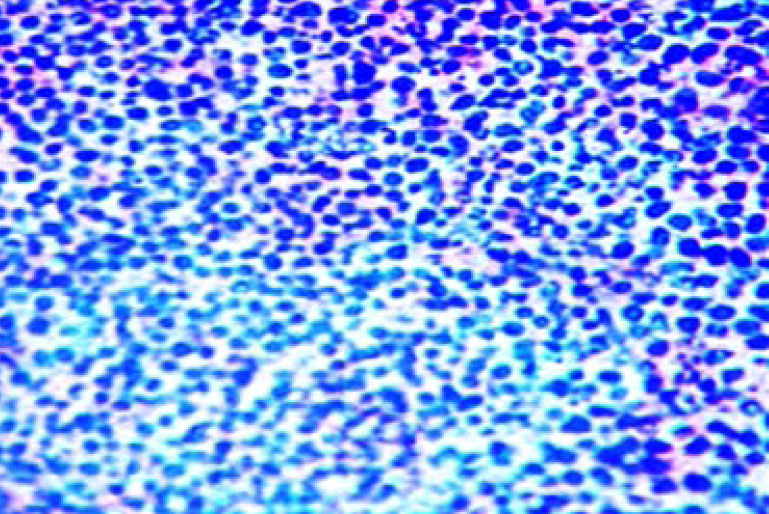
Photomicrograph of retinoblastoma showing sheet of small blue cells with scant cytoplasm, hyperchromatic nuclei and scanty stroma. (Haematoxylin and Eosin stains, X40).

**Figure 3:**
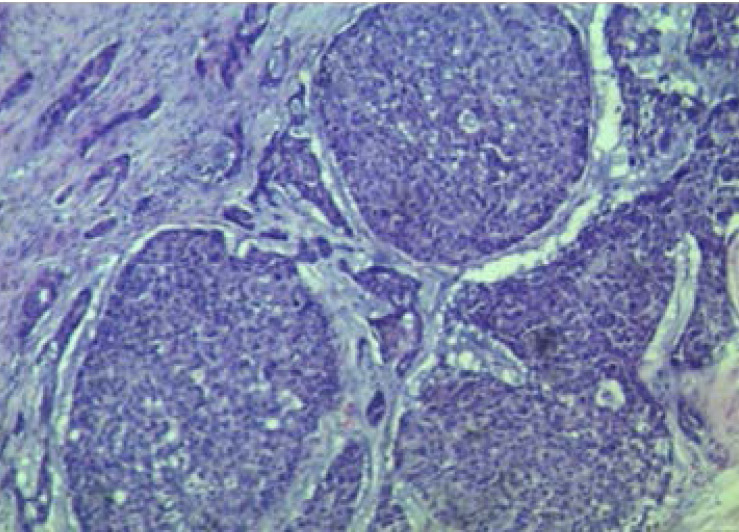
Photomicrograph showing adenoid cystic carcinoma consisting of solid aggregates of basaloid cells with hyperchromatic nuclei, prominent nucleoli and desmoplastic stroma. Also seen are tubules lined by these cells. (Haematoxylin and Eosin stains, X100).

There are 85 cases of MOTs with squamous cell carcinoma (figure 1) and retinoblastoma (figure 2) accounting for 45.9% (39/85 cases) and 44.7% (38/85 cases) respectively. Other less common MOTs including but not limited to adenoid cystic carcinoma (figure 3) are as shown in Table 2. The peak age of MOTs was in the 3rd decade (20–29 years age group).

**Figure 4:**
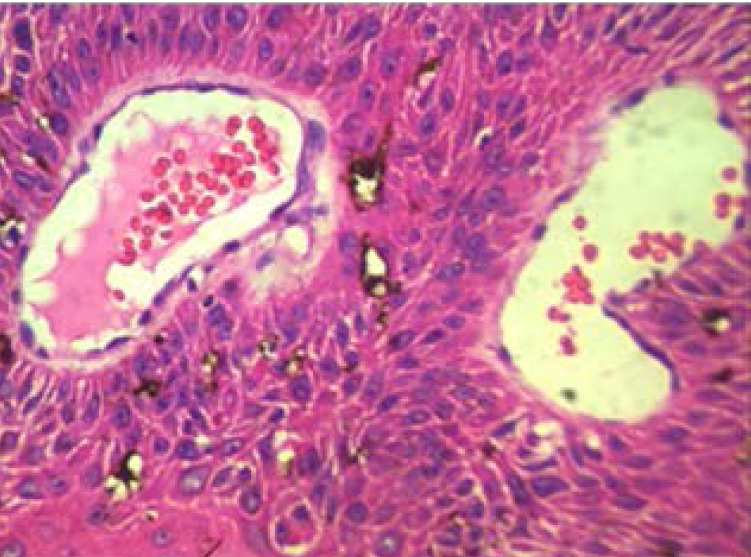
Photomicrograph showing squamous cell papilloma consisting of fibrovascular core surrounded by stratified squamous epithelium. (Haematoxylin and Eosin stains, X100).

Squamous cell papilloma (figure 4) was the most common cause of BOTs seen in this study. It accounted for 31.8% (21/66cases) of cases of benign orbito-ocular tumours. This was followed in decreasing order of frequency by pyogenic granuloma (21%) and lipoma (12.1%) as shown in table 3. Other less common causes of primary benign orbito-ocular tumours are as shown in Table 3.

**Figure 5:**
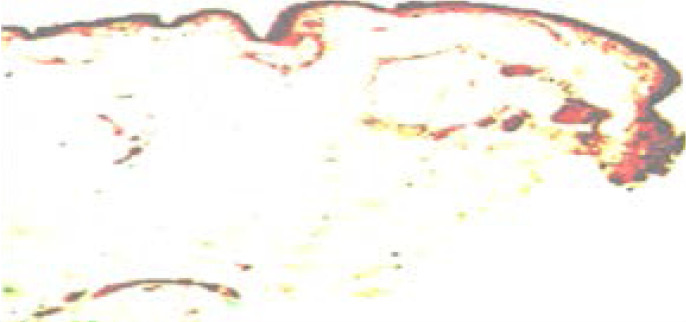
Photomicrograph of pterygium showing elastotic degeneration, prominent congested vessels, and overlying attenuated stratified squamous epithelium. (Haematoxylin and Eosin stains, X100).

Degenerative orbital lesion was secondary to pterygium (figure 5) in 100% of cases (26/26) as shown in Table 4. The 5^th^ to 7^th^ decade accounted for most (11/24) cases of this lesion.

**Figure 6:**
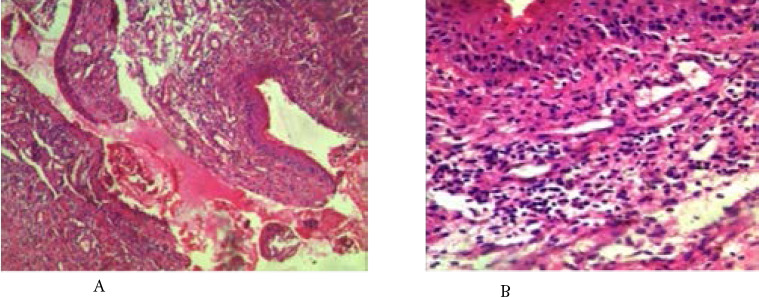
Photomicrographs of conjunctivitis showing infiltrate of inflammatory cells. The stratified squamous epithelium shows reactive hyperplasia. (Haematoxylin and Eosin stains, (a) X 40 and (b) X100).

Chronic panophthalmitis was the most common infective and inflammatory orbito-ocular lesion observed in this study. It accounted for 52.3% (11/21) of cases in this category while hyperplastic inflammatory polyp (1/21) was the least cause of infective and inflammatory orbito-ocular lesions observed in this study. Other causes of infective and inflammatory orbito-ocular lesions are shown in table 4. See photomicrograph of conjunctivitis in figure 6.

Ophthalmic cysts and premalignant lesions accounted for 8% and 5.9% of all orbito-ocular lesions in this study. Other less common orbito-ocular lesions are as shown in table 4.

The conjunctiva (58.1%) and the lacrimal gland (0.40%) are the most and least common sites for orbito-ocular biopsies as shown in Table 5. The site distribution of orbito-ocular biopsies are as shown Table 5.

**Table 5:** Showing frequency distribution of the sites of orbito-ocular lesions

Site of ophthalmic biopsies	Frequency	Percentage (%)
Conjunctiva	137	58.10
Eye ball	61	25.80
Eye lid	28	11.90
Orbit	4	1.69
Lacrimal gland	1	0.40
Not given	5	2.10

Total	236	100.00

## Discussion

Orbito-ocular diseases are an important cause of morbidity-mortality and constitute a major health challenge in sub-Saharan Africa.^3^, ^4^, ^13^ Pathological lesions that ranges from the non-neoplastic (trauma, degenerative, inflammatory and infective) to the neoplastic lesions can affect any of the different constituents of the orbito-ocular system.^2^, ^14^ Variations in the pattern of orbito-ocular diseases are noted especially with regard to age, sex, site and possible aetiological factors.^4^ The University of Benin Teaching Hospital is a referral centre to all other secondary and primary health care facilities within Edo and Delta sub-region and also areas within its catchment area, hence the data generated from ocular and orbital specimens received in the Department of Anatomic Pathology may be regarded as representative indicator of the patterns in the distribution of ocular and orbital lesions in this part of the country.

Orbito-ocular lesions peaked in the 1st decade (0–9 years) in this study. This is consistent with the findings of previous studies in central India by Gupta et al.10 It is however contrary to the findings of Onwubuya et al (north central, Nigeria), Bastola et al (Ahmedabad, Indian) and Chauhan et al (Nepal) who reported a peak incidence in the 4th decade.^4^, ^15^, ^16^ This disparity may be related to the sample sizes of the respective studies, while this study (237 cases) and that of Gupta et al (488 cases) had a higher sample size respectively in comparison to the latter studies whose sample sizes ranged from 66 to 100 cases.^4^,^10^, ^15^, ^16^ Aligbe et al and Anunobi et al reported that the majority of orbito-ocular lesions were found in children less than or equal to 15 years of age while Bekibele et al reported that over 50% of these lesions were observed in children and young adult less than 20 years of age.^13^, ^17^, ^18^ This is consistent with the findings of this study that noted a preponderance of orbito-ocular lesions in individuals less than 20 years of age. The mean age for those with orbito-ocular lesions in this study was in the 3rd decade. This is a decade less than what previous studies done in Nigeria had reported.^4^, ^19^, ^20^ Again, this disparity may be related to samples size which is between 66 to 148 and this is less than the sample size in the present series. We also observed that orbito-ocular lesions have a wide age range spanning infancy (6 months) through old age (10^th^ decade). This finding is comparatively similar to what was previously observed by Onwubuya et al (2–70 years), Anunobil et al (20 days-79 years), Charles et al (1month – 84years) and Aghogho et al (2 months – 84 years).^4^, ^17^, ^19^,^20^ Previous studies in Nigeria and Indian overwhelmingly reported that more males were affected by orbito-ocular lesions in comparison to the females.^4^, ^10^, ^15^–^21^ This is contrary to our own finding that reported a male to female ratio that was slightly in favour of the females.^4^, ^10^, ^15^–^21^

Malignant orbito-ocular tumour was the most common indication for ophthalmic biopsies in this study accounting for approximately one-third (36.00%) of ophthalmic biopsies. This is consistent with the findings by Aligbe et al, Charles et al and Bekibele et al who independently reported that malignant opthalmic lesions were the most common indications for ophthalmic biopsies.^13^, ^18^,^20^ A value of 36.00% of malignant orbito-ocular tumour fall within the range of malignant orbito-ocular tumour (31.4% - 55.11%) from these aforementioned previous studies.13, 18, 20 Squamous cell carcinoma (SCC) was the most common malignant lesion in this study. This finding is consistent with that of previous workers.^4^, ^20^–^24^ Retinoblastoma was the next most frequent ophthalmic malignant lesion following SCC in this study. This is consistent with the findings of Malik et al (Sudan) and Poso et al (Congo-Kinshasha) who reported that this lesion was next to SCC in frequency.^22^, ^23^ Umar et al and Gupta et al, however reported retinoblastoma to be the most common ophthalmic malignant lesion.^10^, ^25^ Common amongst these findings, including that of this study is that retinoblastoma is the most common malignant childhood tumour.^10^, ^20^, ^22^, ^23^, ^25^

The benign ophthalmic neoplastic lesions were the second most common (26.7%) indication for ophthalmic biopsies in this study. This is consistent with the reports of Charles et al (Benin City) and Ackuaku-Dogbe (Korle-Bu, Ghana) who noted that 27.9 to 29.5% of ophthalmic biopsies were benign ophthalmic neoplastic lesions thus making it the second most common indication for ophthalmic biopsies in these series.^20^, ^24^ Unlike this study, higher frequencies (percentages) of benign ophthalmic neoplastic lesions ranging from 54.4% to 70% have been reported in Nepal and India thus making it the most common indication of ophthalmic lesions in these series.^15^,^16^, ^21^ This study showed that squamous cell papilloma (33.3%) was the most frequent benign ophthalmic lesion, followed by pyogenic granuloma and lipoma. Umar et al, Charles et al and Onwubuya et al had reported previously from their respective studies that squamous cell papilloma (13.6% to 32.58%) was the most common benign neoplastic lesion.^4^, ^20^, ^25^ Gupta et al reported that vascular (angiomatous) tumours were the most common (44.7%) benign neoplastic lesion, unlike this study that noted that vascular tumours (pyogenic granuloma) occurred at a lesser percentage (23.8%).^10^ Gupta et al and Charles et al reported that the benign tumour of adipocytes (lipoma) accounted for 2.6% representing the least common tumour and 12.9% representing the second most common tumour respectively of benign ophthalmic neoplastic lesions.^10^, ^20^ The percentage (12.7%) of lipoma in this study is comparatively similar to that of Charles et al from the same environment, however unlike Charles et al observation, it was the third most common lesion in this study.20 We observed that degenerative ophthalmic lesion was the third most common (11%) indication for ophthalmic biopsies. Ackuaku (Korle-Bu, Ghana), Gupta et al (India), Onwubuya et al (Keffi, North Central, Nigeria) and Charles et al (Benin City, Nigeria) reported that degenerative lesion accounted for 1.8% to 10.5% of ophthalmic biopsies.^4^, ^10^, ^20^, ^24^ Pterygium accounted for all the cases of degenerative lesion in the report of Charles et al and cases were seen in the young and middle ages.20 This is similar to the findings of this study. A high lifetime exposure to UV (ultraviolet) rays had been shown to be strongly associated with the development of pterygium.^26^, ^27^ In the same vain, genetic alterations of proto-oncogenes such as K-RAS (Kirsten-Ras), alterations in the expression of tumour suppressor genes (p53 or p63), elaboration of several cytokines (including growth factors and growth factor receptors) by UV radiation mediated expression and a high prevalence of human papilloma viruses (HPVs) in pterygium tissue samples have been found in association with the development of this lesion.^27^ To this end, pterygium may actually represent a proliferative lesion rather than a degenerative lesion.^27^

The infective and inflammatory ophthalmic lesion was the 4th most common (9%) indication for ophthalmic biopsies. Its value falls within the range of inflammatory ophthalmic lesion (6.06 to 24.8%) with respect to the indication for ophthalmic biopsies as reported from previous studies.^4^, ^10^, ^20^, ^21^ Chronic panophthalmitis was the most common infective and inflammatory lesion observed in this study. This is contrary to the findings of Charles et al who reported that inflammatory lesions of the conjunctiva was the most prevalent cause of inflammatory ophthalmic lesion.^20^ However in this study it was among the least indications of inflammatory ophthalmic biopsies. Chalazion accounted for 1.27% of ophthalmic biopsies in this study. This is comparatively similar to the findings of Bastola et al who reported that this lesion accounted for 1% of ophthalmic biopsies.^15^

Ophthalmic cyst was the 5th most common indication for ophthalmic biopsies accounting for 8.01% of cases. Ophthalmic cyst accounting for a higher percentage (11.89 to 20%) of ophthalmic biopsies had been reported in Nepal and India.^10^, ^15^ Epidermal inclusion cyst (94.7%) was the most prevalent cyst in this study while the lacrimal gland cyst was less common (5.3%) in comparison to the former. Contrary to these findings, Bastola et al reported that dermoid cyst was the most prevalent cystic lesion accounting for 60% of cases in their study.15 This was followed by epidermal inclusion cyst (40%).^15^

Ophthalmic premalignant lesion was the 6th most common (5.91%) indication for ophthalmic biopsies in this study. This falls within the percentage range (3.8–19.6%) of ophthalmic premalignant lesion as observed in previous studies.^4^, ^20^

The conjunctiva was the most common (58.1%) site of ophthalmic biopsies in this study. This is comparatively similar to the findings of previous studies in this environment.^13^, ^20^ It is however contrary to the findings of Anunobi et al and Bastola et al who reported that the conjunctiva was the second most common site for ophthalmic biopsies.^15^, ^17^ They reported that conjunctiva biopsies accounted for 20.1 – 22.0% of ophthalmic biopsies.^15^, ^17^ In this study, intraocular (eyeball) biopsies were the second most common site (25.8%) of ophthalmic biopsies. This is comparatively similar to the findings of previous works in this environment.^13^, ^20^ This finding is in contrast to a previous report from Lagos by Anunobi et al who reported that the eyeball was the most common site for ophthalmic biopsies.^17^ The eye lid was the third most common site (11.9%) of ophthalmic biopsies in this study. This finding is consistent with that of Aligbe et al and Charles et al from the same environment.^13^, ^20^ Contrary to the finding of this study, previous studies from Nepal and India have reported that the eyelid was the most common site of ophthalmic biopsies accounting for 33.6–57.0% of ophthalmic biopsies.^10^, ^15^ The surge in cystic eyelid lesion (dermoid cyst and epidermal inclusion cyst) and Rhinosporidiosis for the Nepal and India studies respectively accounted for this site (eyelid) being the most common site in these studies.^10^, ^15^ The eyeball, the conjunctiva and the eye lid constitute the vast majority of ophthalmic biopsies. This is consistent with the findings of previous researches.^10^, ^13^, ^15^, ^17^, ^20^

There is paucity of published histopathological data on the widespread variation of ophthalmic biopsies and by extension ophthalmic lesions in Nigeria. This has greatly reduced the extent to which the observations of this study in comparison to others would have adequately helped to draw a conclusion on ophthalmic biopsies in Nigeria. This is a limitation of this study.

## Conclusion

There exists a wide array of orbito-ocular lesions for which ophthalmic biopsies were performed for histopathologic diagnosis. This research gave the spectrum and relative frequencies of ophthalmic biopsies and by extension ophthalmic lesions/diseases in Edo State and its environs. This would increase the awareness of ophthalmologist and pathologists to the existing spectrum of orbito-ocular lesions in this region and at the same time improve their diagnostic judgement on these lesions.
